# Iodine Concentration in the Breast Milk and Urine as Biomarkers of Iodine Nutritional Status of Lactating Women and Breastfed Infants in Taiwan

**DOI:** 10.3390/nu15194125

**Published:** 2023-09-25

**Authors:** Chun-Jui Huang, Jia-Zhen Li, Chii-Min Hwu, Harn-Shen Chen, Fan-Fen Wang, Chang-Ching Yeh, Chen-Chang Yang

**Affiliations:** 1 Division of Endocrinology and Metabolism, Department of Medicine, Taipei Veterans General Hospital, Taipei 11217, Taiwan; cjhuang3@vghtpe.gov.tw (C.-J.H.);; 2 School of Medicine, College of Medicine, National Yang Ming Chiao Tung University, Taipei 11221, Taiwan; 3 Institute of Food Safety and Health Risk Assessment, College of Pharmaceutical Sciences, National Yang Ming Chiao Tung University, Taipei 112304, Taiwan; 4 Department of Medicine, Yangming Branch, Taipei City Hospital, Taipei 11146, Taiwan; 5 Department of Obstetrics & Gynecology, Taipei Veterans General Hospital, Taipei 11217, Taiwan; 6 Department of Obstetrics & Gynecology, Faculty of Medicine, School of Medicine, National Yang Ming Chiao Tung University, Taipei 11221, Taiwan; 7 Department of Nurse-Midwifery and Women Health, College of Nursing, National Taipei University of Nursing and Health Sciences, Taipei 11219, Taiwan; 8 Institute of Public Health, School of Medicine, National Yang Ming Chiao Tung University, Taipei 11221, Taiwan; 9 Institute of Environmental & Occupational Health Sciences, School of Medicine, National Yang Ming Chiao Tung University, Taipei 11221, Taiwan; 10 Department of Occupational Medicine and Clinical Toxicology, Taipei Veterans General Hospital, Taipei 11217, Taiwan

**Keywords:** breast milk, inductively coupled plasma mass spectrometry, iodine, lactation, thyroid, urinary iodine concentration

## Abstract

Breast milk iodine concentration (BMIC) can be different when median urinary iodine concentration (UIC) is similar. The BMIC, UIC/creatinine (Cr), estimated 24-h urinary iodine excretion (24-h UIE) of lactating women in Taiwan is unknown. This study enrolled lactating women from Taipei Veterans General Hospital (August 2021–February 2023). Each participant provided a random spot urine sample, two breast milk samples, a blood sample, and completed a food frequency questionnaire on the same day. Iodine measurement was performed by inductively coupled plasma mass spectrometry. The median UIC of the enrolled 71 women was 91.1 μg/L, indicating insufficient iodine status; however, the median BMIC was 166.6 μg/L and this suggested that the amount of iodine delivered through breast milk was adequate for the breastfed infants. BMIC was correlated with UIC/Cr and 24-h UIE (both r_s_ = 0.49) but not with UIC (r_s_ = 0.18) or thyroid stimulating hormone (r_s_ = 0.07). Women who did not consume dairy products (adjusted odds ratio: 24.41, 95% confidence interval: 1.26–471.2) and multivitamins (adjusted odds ratio: 8.26, 95% confidence interval: 1.76–38.79) were at increased odds for having lower BMIC. The results suggest that measuring maternal UIC alone may not be sufficient, as BMIC, UIC/Cr, and 24-h UIE are all important biomarkers. Ingestion of dairy products and multivitamins were independently associated with BMIC.

## 1. Introduction

Iodine is especially vital in pregnancy and early life to ensure the fetus’s and infant’s growth and neurodevelopment [1,2]. The World Health Organization recommended a daily intake of 250 μg iodine for pregnant and lactating women, which is higher than that for general adults who need 150 μg per day [3]. The current standard to assess iodine nutritional status in both lactating mothers and breastfed infants is to use the median urinary iodine concentration (UIC), where values ≥100 μg/L indicate iodine sufficiency [3,4]. This value is much lower than the recommended dietary allowances because iodine is secreted into breast milk, unlike the usual condition when 90% of dietary iodine is excreted into the urine [4,5,6,7].

In order to determine infants’ iodine status, assessing their median UIC is important but this may be compromised by difficulties in sample collection [3,4]. According to a recent meta-analysis, the iodine nutritional status of breastfed infants can be reflected by the median breast median iodine concentration (BMIC) [8]. Assessing maternal UIC alone can be misleading because studies have shown that BMIC can be completely different in exclusively breastfeeding women with similar maternal UIC and that populations with maternal median UIC ≥100 μg/L may still have inadequate BMIC values [9,10]. The correlation between BMIC and UIC has not always been consistent in the literature [11,12,13]. While UIC may be affected by maternal fluid intake, maternal UIC adjusted by creatinine (UIC/Cr) or estimated 24-h urine iodine excretion (24-h UIE) has been proposed to be better predictors of BMIC, but relevant literature is limited [12,14,15].

In our previous study evaluating iodine status of lactating women in Taiwan, the maternal median UIC of those who fed their babies with more breast milk (>50%) was lower than the other two groups who fed their babies with 50% or less than 50% of breast milk (UIC: 86.1, 118.2, and 154.1 μg/L for breast milk >50%, 50%, and <50%, *p* = 0.004) [16]. Infant diet composition (breastfed percentage) was significantly associated with maternal UIC < 100 μg/L in multivariable analysis. The possible loss of iodine from breastfeeding may contribute to the result, but unfortunately, BMIC was not measured in that study [16]. The correlation between maternal UIC, UIC/Cr, 24-h UIE, and BMIC is uncertain in Taiwan, an area with borderline iodine adequacy. The study aims to determine the BMIC in lactating women in Taiwan and to explore the association between maternal UIC, UIC/Cr, 24-h UIE, dietary habits and thyroid function with BMIC.

## 2. Materials and Methods

### 2.1. Study Design and Sample Collection

This cross-sectional, hospital-based study enrolled lactating women aged 20 years or above who visited Taipei Veterans General Hospital for routine checkups during the postpartum 15- to 90-day period, from August 2021 to February 2023. Women who were diagnosed with hypothyroidism, hyperthyroidism, and those who were taking anti-thyroid drugs or levothyroxine were excluded. All samples were collected on the same day, including a random spot urine sample for measurement of UIC and Cr, two random breast milk samples (one from each breast) for determination of BMIC, and a blood sample for measurement of thyroid stimulating hormone (TSH), free T4, anti-thyroglobulin antibodies (aTG), and anti-thyroid peroxidase antibodies (aTPO). A simple food frequency questionnaire (FFQ) was completed on the day of sample collection. This study was approved by the local Institutional Review Board (IRB No: 2021-06-006B). Written informed consent was obtained from all participants prior to enrollment.

### 2.2. Iodine and Thyroid Function Measurements

All samples were kept frozen at −20 °C after being collected. Urine samples were thawed at room temperature and breast milk samples were heated in the oven at 40 °C for 20 min before analysis. Urinary and breast milk iodine measurements were performed using an Agilent 7700 Series inductively coupled plasma mass spectrometry system as previously described [17,18]. Whole milk powder reference material (National Institute of Standards and Technology, NIST, 1549 non-fat milk powder purchased from NIST, Washington, DC., USA), urine reference material (REF 8847, REF 8849 purchased from RECIPE Co. Munich, Germany), and human urine samples from the Ensuring the Quality of Urinary Iodine Procedures program (Centers for Disease Control and Prevention, Washington DC, USA) were measured in each run for quality control. The limit of detection for urinary iodine measurement was 1.0 μg/L and for breast milk analysis was 0.78 μg/L [17,18].

Serum TSH, free T4, aTG, and aTPO were measured by an electrochemiluminescence immunoassay (cobas e 801; Roche Diagnostics GmbH., Mannheim, Germany). The reference value of aTG and aTPO is <115 IU/mL and <60 U/mL, respectively. The reference value of TSH is 0.27–4.20 uIU/mL and free T4 is 0.93–1.7 ng/dL. The department of pathology and laboratory medicine of the hospital was commissioned to test for urine Cr (Roche Cobas c 701, Roche Diagnostics GmbH., Mannheim, Germany). The estimated 24-h UIE was calculated as: UIC/Cr (μg/g) × predicted 24-h Cr (g/day) [19,20,21]. The predicted 24-h Cr (g/day) = 0.00163 × [140 − age (years)] × [weight (kg)^1.5^ × height (cm)^0.5^] × [1.429 − 0.0198 × body mass index (kg/m^2^)]/1000 [20,21].

### 2.3. Food Frequency Questionnaire

The FFQ was similar to that which had been previously described [16]. It contained questions surveying the frequency of consumption of iodine-containing food types, whether it was 1, 3, 5, 7 days per week, or never. The food types included (1) seaweeds, (2) fish, (3) seafood (except fish), (4) dairy foods, (5) multivitamins, and (6) iodized salt. Participants viewed an illustrated chart of multivitamins and postpartum nourishment diets to identify the brands. Information on breastfeeding conditions was also collected.

### 2.4. Statistical Analysis

UIC, UIC/Cr, 24-h UIE and BMIC were not normally distributed and were presented as median with inter-quartile range, while the other continuous variables were expressed as mean ± standard deviation. The Mann-Whitney U test was performed for comparison of continuous variables. The categorical variables were presented as numbers with percentages and analyzed by the Spearman’s Chi-square test. Variables with a *p*-value < 0.2 in univariable analysis were further analyzed in multivariable logistic regression models to determine the risk factors for lower median BMIC. In multivariable analysis, the Akaike information criterion was used to compare the goodness-of-fit of different models. The relationship between UIC, UIC/Cr, 24-h UIE, BMIC, and TSH was assessed by the Spearman’s rank correlation test. All data analyses were performed using the SAS software, version 4.9. A two-tailed *p*-value of <0.05 was considered statistically significant. 

## 3. Results

### 3.1. Baseline Characteristics

The characteristics of the study population are shown in Table 1. A total of 71 women, with a mean age of 34.0 ± 4.1 years old (range: 25–44 years), were enrolled at a mean of 44.8 ± 9.4 days postpartum. Among them, 23.9% (*n* = 17) of the women were exclusively breastfeeding and most of them (*n* = 58, 81.7%) were not under a postpartum nourishment diet. 

The consumption frequency of various iodine-containing food types is presented in Table 2. Dairy products were the most frequently consumed food type whereas seafood, fish, and seaweeds were less frequently eaten. The consumption frequency of multivitamins varied, with 28.2% (*n* = 20) of the women taking a multivitamin every day, some ingesting it occasionally (19.7%, *n* = 14), while the remaining 52.1% (*n* = 37) never took multivitamins.

### 3.2. Iodine Concentration Analysis

The median UIC, UIC/Cr and 24-h UIE of the study population were 91.1 μg/L (IQR: 42.6–145.0 μg/L), 132.8 μg/g (IQR: 86.7–214.1 μg/g) and 138.0 μg/day (IQR: 88.2–234.2 μg/day), respectively (Table 1). The overall median BMIC was 166.6 μg/L (IQR: 116.9–256.9 μg/L) and the results were similar between the right and left breast (right: 165.8 μg/L, IQR: 112.3–266.7 μg/L; left: 177.9 μg/L, IQR: 112.8–278.3 μg/L). No variables were significantly associated with lower UIC (Supplementary Table S1). Variables associated with lower median BMIC <166.6 μg/L were analyzed, and the results showed that lower UIC/Cr (*p* < 0.001), lower 24-h UIE (*p* < 0.001), intake of seafood (*p* = 0.004) and no ingestion of multivitamins (*p* = 0.008) were significantly associated with lower median BMIC (Table 3).

In multivariable logistic regression models, intake of seafood [adjusted odds ratio (OR): 16.0, 13.3, 12.2, 12.1; 95% confidence interval (CI): 2.3–111.4, 2.9–60.7, 2.3–66.2, 2.1–70.9], no ingestion of dairy products (adjusted OR: 24.41, 11.38, 17.07, 20.07; 95% CI: 1.26–471.2, 1.09–119.19, 1.21–240.44, 1.23–328.54) and no intake of multivitamins (adjusted OR: 8.26, 5.22, 5.62, 7.28; 95% CI: 1.76–38.79, 1.47–18.58, 1.44–21.96, 1.74–30.37) were independently associated with BMIC <166.6 μg/L in all models; whereas UIC (adjusted OR: 0.99; 95% CI: 0.98–1.0) in model two, UIC/Cr (adjusted OR: 0.98; 95% CI: 0.97–0.99) in model three and 24-h UIE (adjusted OR: 0.98; 95% CI: 0.97–0.99) in model four were also independent risk factors (Table 4).

Characteristics of lactating women who exclusively breastfed were presented in Table 5. Compared to the women who fed their babies with both breast milk and infant formula, the UIC/Cr values of the exclusively breastfeeding women (103.0 μg/g vs. 145.9 μg/g, *p* = 0.04) were significantly lower, but the other variables, including UIC, 24-h UIE, and BMIC were not significantly different.

### 3.3. Correlation between UIC, BMIC and Thyroid Function

**Figure 1:**
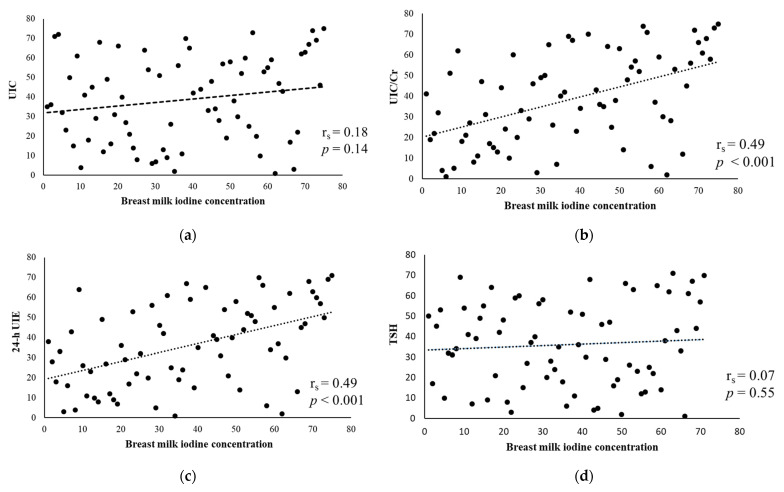
Scatter plots of the correlation between BMIC and (**a**) UIC, urinary iodine concentration; (**b**) UIC/Cr, urinary iodine concentration/creatinine; (**c**) 24-h UIE, 24-h urinary iodine excretion; (**d**) TSH, thyroid stimulating hormone.

The result of the correlation analysis is presented in Figure 1. BMIC was correlated with UIC/Cr (r_s_ = 0.49, *p* < 0.001), Figure 1b, and 24-h UIE (r_s_ = 0.49, *p* < 0.001), Figure 1c, but not correlated with UIC (r_s_ = 0.18, *p* = 0.14), Figure 1a and TSH (r_s_ = 0.07, *p* = 0.55), Figure 1d.

## 4. Discussion

This is the first time data on maternal UIC, UIC/Cr, 24-h UIC and BMIC were reported in an area with borderline iodine adequacy. The median UIC, UIC/Cr, 24-h UIE, and BMIC were 91.1 μg/L, 132.8 μg/g, 138.0 μg/day and 166.6 μg/L, respectively. The median UIC value was <100 μg/L in the study population, and this indicates insufficient maternal iodine status [3,4]. However, the median BMIC was ≥100 µg/L and this suggests that the iodine supply in the breast milk is sufficient for the breastfed infants [10,22,23,24,25,26]. The values of UIC/Cr and 24-h UIE were higher than the UIC value and better correlated with BMIC. There was no correlation between TSH and BMIC, and the maternal UIC value in the study population was considered sufficient to maintain euthyroidism [27,28]. Risk factors related to lower median BMIC included intake of seafood, no ingestion of dairy products and multivitamins.

Measuring infant UIC is the gold standard for assessing infant iodine status, but this measurement can be biased by difficulties in sample collection [3,4]. For exclusively breastfed infants, the supply of iodine relies solely on the iodine in breast milk. A significant correlation between BMIC and infant UIC has been demonstrated and this makes BMIC a promising biomarker [22,29,30]. Based on the average lactation volume of 670 mL in the Asian population, the estimated average iodine amount in breast milk of the study population is 111.6 μg/day (IQR: 78.3–172.1), which is sufficient for infants aged 0–6 months in general (adequate intake: 110 μg/day) [3,31]. In this study, the median BMIC of the exclusively breastfed infant group was 138.3 µg/L and the calculated infant’s iodine intake from breast milk was 121.7 μg per day, based on the average breast milk intake of exclusively breastfed infants of 880 mL/day [3,31]. Assuming that 90% of the ingested iodine is secreted into breast milk or urine, the estimated maternal iodine intake is 277 μg/day [(24-h UIE 138.0 μg/day + iodine amount in breast milk 111.6 μg/day) ÷ 90%], which is higher than the recommended intake of 250 μg/day for lactating women [3]. Although the median UIC value suggests insufficient maternal iodine status, the estimated maternal iodine intake meets the current recommendation after considering the amount of iodine secreted into breast milk.

Studies have shown that the UIC in spot urine samples can be largely influenced by the amount of fluid intake, and the values present high day-to-day variability [14,15,32]. The 24-h UIE has been regarded as the gold standard, but the time required and inconvenience in sample collection limit its widespread use in population studies [32,33,34]. The UIC adjusted by Cr in spot urine samples, or the estimated 24-h UIE, emerged as alternatives to overcome these limitations [14,15]. It has been shown that UIC/Cr can more precisely reflect iodine intake in non-lactating subjects [33,35]. In breastfeeding women, BMIC can be better predicted by maternal UIC/Cr and 24-h UIE, while UIC generally has no correlation with BMIC [11,12,13]. The result of the current study implies that the iodine supply to the breastfed infants is more reliably evaluated by BMIC, maternal UIC/Cr, and estimated 24-h UIE, but not by maternal UIC value.

In areas with sufficient iodine intake, the median BMIC and UIC can both be ≥100 μg/L; in places with low iodine intake, the BMIC and UIC can both be <100 μg/L rendering the breastfed infants at risk for iodine deficiency. In an area with borderline iodine adequacy such as Taiwan, the median BMIC and UIC values can be discrepant [9,11,12,13,15,23,24,30,36,37,38]. Maternal fluid intake is one of the reasons; another possible explanation is the preferential secretion of iodine into breast milk when iodine intake is at the border for adequacy [9,15]. According to the Nutrition and Health Survey in Taiwan, the median UIC was 100 μg/L for adults over 19 years old in 2005–2008, 96 μg/L for people aged over 6 years in 2013, and 104 μg/L for adults over 19 years old in 2017–2020 [39,40,41]. The change of salt iodization policy from mandatory to voluntary after Taiwan joined the World Trade Organization in 2002 contributed to the mildly deficient or borderline adequate iodine status in the past 20 years [39,40,41]. To ensure adequate iodine nutrition to the breastfed infants, absorbed dietary iodine can be partitioned more into breast milk under the regulation of prolactin and other hormones through the sodium-iodide symporter [42,43]. At the expense of maternal iodine reserve and lower UIC value, this compensates for the slightly inadequate maternal iodine status and provides sufficient iodine supply to the breastfed infants [42].

Universal salt iodization has been recommended since 1993 [44]; nevertheless, most of the available salts in the market in Taiwan are non-iodized [45]. In the current study, only half of the women used iodized salt, and the common phenomenon of eating non- self-prepared foods made salt an unstable source of iodine nutrition. Iodine-containing foods and supplements were therefore very important. In this study, women who did not ingest dairy products had an increased chance of having a lower BMIC, and this finding was compatible with other studies that demonstrated lower BMIC in women with less intake of dairy products [38,46]. In a survey measuring iodine content in 76 samples of milk types in Taiwan, the iodine concentrations of whole milk, low-fat milk, flavored milk drinks, and milk alternative drinks were determined to be 210.4 μg/L, 263.2 μg/L, 100.0 μg/L, and 65.6 μg/L on average [47]. Drinking a carton of whole milk or low-fat milk (400 c.c.) would provide one-third of the daily iodine requirement for lactating women, and this should be regarded as an important dietary source of iodine nutrition.

Interestingly, women who ingested seafood were also at increased odds for lower BMIC, which is in contrast to the common understanding that seafood is a type of iodine-rich food. It is possible that women did not ingest seafood in amounts sufficient to supply much iodine and it is also reasonable to think that when women ingest seafood in larger amounts, the intake frequency of other iodine-containing food types became less. Unfortunately, the amount of iodine ingested from seafood could not be determined in the current study because there was no information about the portion size in the FFQ, and the iodine content in seafood is still unknown in Taiwan.

In order to provide sufficient iodine nutrition to breastfed infants, the American Thyroid Association has recommended that lactating women, similar to pregnant women, should ingest iodine supplements containing 150 μg iodine per day [48]. Studies have demonstrated that BMIC tends to be higher in women with habitual intake of iodine supplement [15,36]. Among the women who took multivitamins in this study, 70.4% of them took the brands that contained iodine, which should result in higher iodine levels in breast milk. Non-consumption of multivitamins was an independent predictor of lower median BMIC in the study population, and based on this result, iodine supplementation during lactation could be recommended.

It is part of the traditional Chinese culture that women receive specialized postpartum care and ingest a postpartum nourishment diet for 15–45 days after delivery [49,50]. In our previous study evaluating the iodine status of lactating women, the time of enrollment was 13.6 days after delivery, and 74% of the women were ingesting a postpartum nourishment diet, which was significantly higher than 18.3% in this study when women were enrolled at 44.8 days after delivery [16]. Fish and seafood are not common food types in daily life in Taiwan but appear very often in the postpartum nourishment diet. All these factors taken into account together contributed to the differences in iodine intake in women in these two studies, and thus the difference in median UIC (120.4 μg/L vs. 91.1 μg/L) [16].

There are several limitations. First, the infant’s UIC and thyroid function were not measured due to difficulties in sample collection. Second, although the present FFQ could not be used to calculate the actual amount of iodine ingested, it did, to some extent, reflect the iodine intake of the studied women. Third, this study was conducted in a tertiary referral center in the capital city and could not be generalized to other areas in Taiwan. We also did not record the time of the collection of the random urine and breast milk samples and their proximity to the last meal and breastfeeding. We acknowledge that circadian rhythm, post-meal peaks and downward trend post-breastfeeding may be potential sources for unknown bias to influence the iodine concentration in urine and breast milk [51,52].

## 5. Conclusions

The results of the current study suggest that BMIC, UIC/Cr, and 24-h UIE are all important biomarkers to assess iodine nutritional status in lactating women. The iodine supply to the breastfed infants could not be accurately evaluated by measuring maternal UIC alone. Ingestion of dairy products and multivitamins was independently associated with BMIC in lactating women in Taiwan.

## Figures and Tables

**Table 1:** Characteristics of the study population.

Variables	Mean ± SD or Number (%)
Age, years	34.0 ± 4.1
Height, cm	161.3 ± 4.9
Weight, kg	63.9 ± 9.8
BMI, kg/m^2^	24.6 ± 3.5
TSH, uIU/mL	1.6 ± 0.9
Free T4, ng/dL	1.1 ± 0.2
Positive aTG, *n* (%)	1 (1.4)
Positive aTPO, *n* (%)	0 (0)
Time of postpartum, days	44.8 ± 9.4
Infant birth body weight, g	3012.9 ± 436.4
UIC, μg/L	91.1 (42.6–145.0)
UIC/Cr, μg/g	132.8 (86.7–214.1)
24-h UIE, μg/d	138.0 (88.2–234.2)
BMIC, μg/L	166.6 (116.9–256.9)
Right breast BMIC, μg/L	165.8 (112.3–266.7)
Left breast BMIC, μg/L	177.9 (112.8–278.3)
Educational status, *n* (%)	
University or below	58 (81.7)
Master or above	13 (18.3)
Parity ≥ 2, *n* (%)	30 (42.3)
Positive miscarriage history, *n* (%)	27 (38.0)
Under postpartum nourishment diet, *n* (%)	13 (18.3))
Exclusively breastfeeding, *n* (%)	17 (23.9)

UIC, UIC/Cr, 24-h UIE, and BMIC were presented using median and interquartile range. Other variables were either presented using mean and standard deviation or as number and percentage. aTG, anti-thyroglobulin antibodies; aTPO, and anti-thyroid peroxidase antibodies; BMI, body mass index; BMIC, breast milk iodine concentration; TSH, thyroid stimulating hormone; UIC, urinary iodine concentration; UIC/Cr, urinary iodine concentration/creatinine; 24-h UIE, 24-h urinary iodine excretion.

**Table 2:** The result of FFQ: number of days per week in consuming various foods.

Food Type (*n* %)	0 Day (%)	1 Days (%)	3 Days (%)	5 Days (%)	7 Days (%)
Seaweed	29 (40.8)	37 (52.1)	4 (5.6)	1 (1.4)	0 (0.0)
Fish	12 (16.9)	29 (40.8)	21 (29.6)	5 (7.0)	4 (5.6)
Seafood (except fish)	29 (40.8)	31 (43.7)	9 (12.7)	2 (2.8)	0 (0.0)
Dairy products	8 (11.3)	17 (23.9)	14 (19.7)	8 (11.3)	24 (33.8)
Multivitamins	37 (52.1)	7 (9.9)	4 (5.6)	3 (4.2)	20 (28.2)

Data was presented as median and interquartile range.

**Table 3:** The proportion of women with BMIC ≥ or < 166.6 μg/L.

	BMIC, *n* (%)		
	<166.6 μg/L	≥166.6 μg/L	*p*
Age, years	33.2 ± 3.9	34.8 ± 4.3	0.19
Educational status			0.34
University or below (*n* = 58)	27 (46.6)	31(53.4)	
Master or above (*n* = 13)	8 (61.5)	5 (38.5)	
Infant birth body weight, g	3120.8 ± 147.9	2996.6 ± 489.1	0.50
Parity			0.93
First (*n* = 41)	20 (48.8)	21 (51.2)	
Second or above (*n* = 30)	15 (50.0)	15 (50.0)	
Miscarriage history			0.74
Yes (*n* = 27)	14 (51.9)	13 (48.1)	
No (*n* = 44)	21 (47.7)	23 (52.3)	
Postpartum nourishment diet			0.81
Yes (*n* = 13)	6 (46.2)	7(53.8)	
No (*n* = 58)	29 (50.0)	29 (50.0)	
Exclusively breastfed			0.37
Yes (*n* = 17)	10 (58.8)	7 (41.2)	
No (*n* = 54)	25 (46.3)	29 (53.7)	
Master or above	13 (18.3)		
Salt intake			0.33
Iodized (*n* = 39)	17 (43.6)	22 (56.4)	
Non-iodized (*n* = 14)	8 (57.1)	6 (42.9)	
Unknown (*n* = 18)	10 (55.6)	8 (44.4)	
Seaweed intake			0.54
Yes (*n* = 42)	22 (52.4)	20 (47.6)	
No (*n* = 29)	13 (44.8)	16 (55.2)	
Fish intake			0.57
Yes (*n* = 59)	30 (50.8)	29 (49.2)	
No (*n* = 12)	5 (41.7)	7 (58.3)	
Seafood intake			0.004
Yes (*n* = 42)	27 (64.3)	15 (35.7)	
No (*n* = 29)	8 (27.6)	21 (72.4)	
Dairy intake			0.13
Yes (*n* = 63)	29 (46.0)	34 (54.0)	
No (*n* = 8)	6 (75.0)	2 (25.0)	
Multivitamins intake			0.008
Yes (*n* = 34)	16 (47.1)	18 (52.9)	
No (*n* = 37)	19 (51.4)	18 (48.6)	
UIC, μg/L	76.9 (35.7–115.7)	111.4 (64.3–164.3)	0.06
UIC/Cr, μg/g	99.8 (66.8–149.0)	187.7 (124.3–250.7)	<0.001
Cr, μg/g	79.6 (40.8–139.4)	60.6 (34.9–94.3)	0.17
24-h UIE, μg/d	103.5 (67.1–146.0)	197.8 (135.0–271.3)	<0.001

UIC, UIC/Cr, Cr, and 24-h UIE were presented using median and interquartile range. Other variables were presented either as mean and standard deviation or as number and percentage. Cr, creatinine; UIC, urinary iodine concentration; UIC/Cr, urinary iodine concentration/creatinine; 24-h UIE, 24-h urinary iodine excretion.

**Table 4:** Multivariable analysis of factors associated with lower median BMIC.

	Model 1		Model 2		Model 3		Model 4	
Variables	OR (95% CI)	*p*	OR (95% CI)	*p*	OR (95% CI)	*p*	OR (95% CI)	*p*
Age	0.85 (0.70–1.02)	0.08	0.86 (0.73–1.01)	0.06	0.88 (0.75–1.50)	0.16	0.86 (0.72–1.03)	0.1
Seafood intake (ref: non-user)	15.97 (2.29–111.44)	0.005	13.35 (2.94–60.68)	<0.001	12.24 (2.26–66.17)	0.004	12.10 (2.07–70.85)	0.006
Dairy intake (ref: user)	24.41 (1.26–471.22)	0.003	11.38 (1.09–119.19)	0.04	17.07 (1.21–240.44)	0.04	20.07 (1.23–328.54)	0.04
Multivitamins intake (ref.: user)	8.26 (1.76–38.79)	0.007	5.22 (1.47–18.58)	0.01	5.62 (1.44–21.96)	0.01	7.28 (1.74–30.36)	0.007
UIC, μg/L	0.99 (0.98–1.00)	0.28	0.99 (0.98–1.00)	0.04				
UIC/Cr, μg/g	1.00 (0.97–1.04)	0.88			0.98 (0.97–0.99)	0.003		
24-h UIE, μg/d	0.98 (0.95–1.01)	0.26					0.98 (0.97–0.99)	0.002

UIC, urinary iodine concentration; UIC/Cr, urinary iodine concentration/creatinine; 24-h UIE, 24-h urinary iodine excretion.

**Table 5:** Characteristics of lactating women who exclusively breastfed.

Variables	Exclusively Breastfeeding (*n* = 17)	Others (*n* = 54)	*p*
Age, years	32.4 ± 3.7	34.6 ± 4.2	0.04
UIC, μg/L	87.7 (59.9–123.7)	94.6 (42.6–171.5)	0.60
UIC/Cr, μg/g	103.0 (64.5–157.9)	145.9 (98.0–230.7)	0.04
24-h UIE, μg/d	91.3 (62.8–173.8)	143.3 (102.0–235.1)	0.11
BMIC, μg/L	138.3 (116.9–210.7)	168.4 (124.6–266.7)	0.35

Age was presented as mean and standard deviation whereas other variables were presented as median and interquartile range. BMIC, breast milk iodine concentration; UIC, urinary iodine concentration; UIC/Cr, urinary iodine concentration/creatinine; 24-h UIE, 24-h urinary iodine excretion.

## Data Availability

Data are available on request from the corresponding author.

## Supplementary Materials

The following supporting information can be downloaded at: https://www.mdpi.com/article/10.3390/nu15194125/s1, Table S1: The proportion of women with UIC ≥ or <100 μg/L.
