# Supplementary material for: Iodine Concentration in the Breast Milk and Urine as Biomarkers of Iodine Nutritional Status of Lactating Women and Breastfed Infants in Taiwan
Source: Nutrients. 2023 Sep 25;15(19):4125. doi: 10.3390/nu15194125 (PMC10574722; doi:10.3390/nu15194125)
Supplement: Supplementary file 1 [file nutrients-15-04125-s001.zip › nutrients-2603810-supplementary.pdf]

**Supplementary Table S1**. The proportion of women with UIC ≥ or < 100 μg/L

| | UIC, n (%) | | |
|---|---|---|---|
| | < 100 μg/L (n=37) | ≥ 100 μg/L (n=34) | *p* |
| **Age, years** | 34.3 ± 3.9 | 33.8 ± 4.4 | 0.70 |
| **Educational status** | | | 0.64 |
| University or below (n = 58) | 31 (53.4) | 27 (46.6) | |
| Master or above (n = 13) | 6 (46.2) | 7 (53.8) | |
| **Infant birth body weight, g** | 3062.5 ± 563.3 | 3052.8 ± 309.4 | 0.65 |
| **Parity** | | | 0.44 |
| First (n = 41) | 23 (56.1) | 18 (43.9) | |
| Second or above (n = 30) | 14 (46.7) | 16 (53.3) | |
| **Miscarriage history** | | | 0.14 |
| Yes (n = 27) | 11 (40.7) | 16 (59.3) | |
| No (n = 44) | 26 (59.1) | 18 (40.9) | |
| **Post-partum nourishment diet** | | | 0.18 |
| Yes (n = 13) | 9 (69.2) | 4 (30.8) | |
| No (n = 58) | 28 (48.3) | 30 (51.7) | |
| **Exclusive breastfeeding** | | | 0.94 |
| Yes (n = 17) | 9 (52.9) | 8 (47.1) | |
| No (n = 54) | 28 (51.9) | 26 (48.1) | |
| **Salt intake** | | | 0.32 |
| Iodized (n = 39) | 19 (48.7) | 20 (51.3) | |
| Non-iodized (n = 14) | 6 (42.9) | 8 (57.1) | |
| Unknown (n = 18) | 12 (66.7) | 6 (33.3) | |
| **Seaweed intake** | | | 0.02 |
| Yes (n = 42) | 17 (40.5) | 25 (59.5) | |
| No (n = 29) | 20 (69.0) | 9 (31.0) | |
| **Fish intake** | | | 0.44 |
| Yes (n = 59) | 32 (54.2) | 27 (45.8) | |
| No (n = 12) | 5 (41.7) | 7 (58.3) | |
| **Seafood intake** | | | 0.68 |
| Yes (n = 42) | 21 (50.0) | 21 (50.0) | |
| No (n = 29) | 16 (55.2) | 13 (44.8) | |
| **Dairy intake** | | | 0.18 |
| Yes (n = 63) | 31(49.2) | 32 (50.8) | |
| No (n = 8) | 6 (75.0) | 2 (25.0) | |
| **Multivitamin intake** | | | 0.42 |
| Yes (n = 34) | 16 (47.1) | 18 (52.9) | |
| No (n = 37) | 21 (56.8) | 16 (43.2) | |

Age and infant birth body weight was presented by mean and standard deviation whereas other variables were presented by number and percentage.
